# In silico designing of a multi-epitope vaccine against *Burkholderia pseudomallei*: reverse vaccinology and immunoinformatics

**DOI:** 10.1186/s43141-022-00379-4

**Published:** 2022-07-11

**Authors:** Muhammad Shahab, Chandni Hayat, Ramin Sikandar, Guojun Zheng, Shahina Akter

**Affiliations:** 1 State Key Laboratory of Chemical Resources Engineering, Beijing University of Chemical Technology, Beijing, 100029 China; 2 Department of Biochemistry, Computational Medicinal Chemistry Laboratory, UCSS, Abdul Wali Khan University, Mardan, Pakistan; 3 Bangladesh Council of Scientific and Industrial Research, Dhaka, 1205 Bangladesh

**Keywords:** *Burkholderia*, Epitope, Vaccine, B cell, T cell, Immunoinformatics

## Abstract

**Background:**

*Burkholderia pseudomallei* is an infectious agent causing severe disease melioidosis resulting in pneumonia, fever, and acute septicemia in humans. *B. pseudomallei* show resistance to drugs. No such FDA-approved vaccine is available against *B. pseudomallei*, and treatment is limited to therapy. Therefore, the scientific study was designed to develop a vaccine for *B. pseudomallei*. The protein sequence of *B. pseudomallei* was retrieved from NCBI. B-cell and T-cell epitopes were identified and further screened for allergenicity, antigenicity docking, and simulation.

**Results:**

Here, in this study, in silico approach was applied to design a multi-epitope subunit vaccine peptide consisting of linear B-cell and T-cell epitopes of proteins considered to be potential novel vaccine candidates. Peptide epitopes were joined by adjuvant and EAAAK, CPGPG, and AAY linkers. This constructed vaccine was subjected to in silico immune simulations by C-ImmSim. The protein construct was cloned into PET28a (+) vector for expression study in *Escherichia coli* using SnapGene.

**Conclusion:**

The designed multi-epitope vaccine was analyzed for its physicochemical, structural, and immunological characteristics, and it was found to be antigenic, soluble, stable, nonallergenic, and have a high affinity to its target receptor. The immune simulation studies were carried out on the C-ImmSim showing increased production of cellular and humoral responses indicating that the constructed vaccine proved effective and able to provoke humoral and cell-mediated response immune responses. In silico study could be a breakthrough in designing effective vaccines to eradicate *B. pseudomallei* globally.

## Background

*Burkholderia pseudomallei* — gram-negative bacteria, cause infectious disease melioidosis in Australia and Asia [1]. It has been discovered by Whitmore in 1911 and associated with “glander-like” disease [2]. Among melioidosis patients, 40% of mortality rates were reported in Thailand and 21% in Australia [3].

A wide range of signs including acute septicemia, pneumonia, chronic localized infection, and fever was identified; it can also damage different organs, such as the liver, lungs, kidney, spleen, skeletal muscles, and prostate glands [4]. *B. pseudomallei* is transmitted by ingestion, inhalation, and skin abrasion [5]. Patients with renal disease, thalassemia, and diabetes reported a high risk of melioidosis [6]. Healthy patients have a significantly low risk of melioidosis. The disease may be contracted by inhalation, cutaneous inoculation, or ingestion [7]. Due to different clinical symptoms and presentations with different culture-based anomalies, it is difficult to treat in clinical settings [8]. Successful drug treatment includes strict regimens of antibiotics such as meropenem and ceftazidime for 14 days. However, the use of co-trimoxazole works for 6 months. But *B. pseudomallei* shows resistance to several antibiotics due to which treatment may be complicated [9]. *B. pseudomallei* show intrinsic resistance towards different other drugs also like meropenem resistance against *B*. *ubonensis* [10], which determines the way of similar resistance in *B. pseudomallei*.

Microorganisms are cultured in conventional vaccinology and used for pathogenic identification, isolation, characterization, reinjection, and inactivation in the host to provoke immune response [11], but these old vaccine methods are expensive and time-consuming and not suitable for some pathogens such as *B. pseudomallei*. Improvement in biology systems, proteomics, genomics, and DNA sequencing represents better understanding for estimation of pathogenic organisms for vaccine design. Some methods are cost-effective and have good accuracy and can be applied to a microorganism with the best results [12–14]. Immunoinformatics approaches are used for the determination of vaccine design [15, 16]. In vaccine design, suitable protein selection is important. Protein which is virulent, highly antigenic, and non-homologous for humans can be used to increase efficacy [17].

Different vaccines on basis of epitope predictions formed pathogens in humans, such as epitope-based vaccine development against *Plasmodium vivax* (AMA-1) [18]. Recently, epitope base candidate is against *Acinetobacter baumannii* in mice [19]. B-cell epitopes prediction is against *Trypasonoma vivax* [20]. Different vaccines are developed against human pathogens such as the *Marburgvirus* [21], Ebola virus [22], Mokola Rabies virus [23], and Crimean-Congo hemorrhagic virus [24]. However, B-cell and T-cell epitopes were predicated against SARS-CoV-2 by Ziwei et al., and experimentally checked, showing top immunogenic response in mice model [25]. Keeping in mind the importance of the multi-epitope vaccine, we design a more effective, safe, and thermodynamically stable epitope base vaccine design for *B. pseudomallei* to elicit an innate and adaptive immune response. We used the immunoinformatics approach for vaccine designing, to select nonoverlapping, nonallergic, and topmost antigenic epitopes. Constructed vaccine docked with TL4, and for estimation of effectiveness and stability, simulation was performed.

## Methods

### Retrieval of sequence

**Fig. 1:**
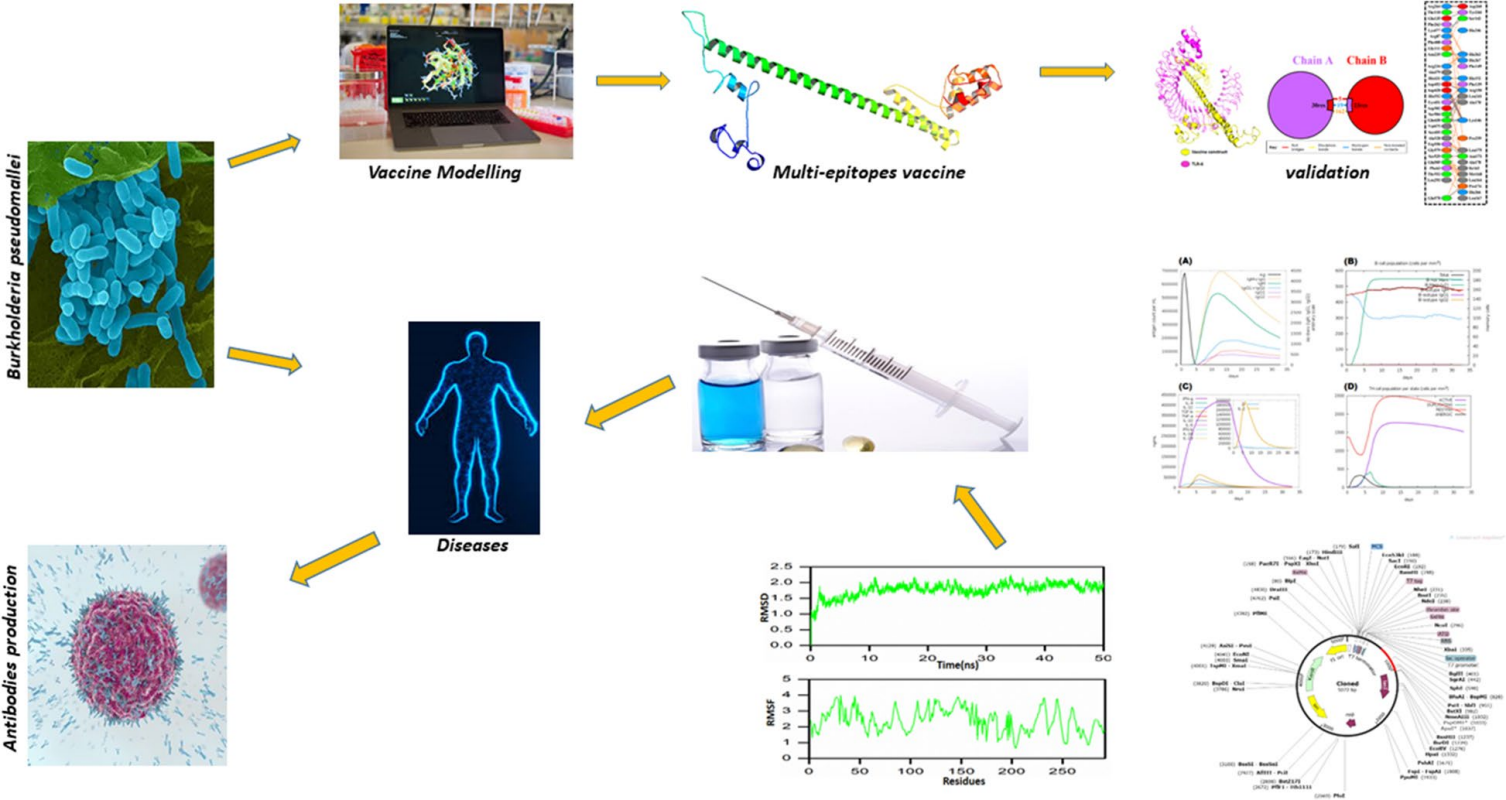
Experimental design for in silico analyses of vaccine design

The protocol for designing a vaccine against *B. pseudomallei* was illustrated in Fig. 1. The research was initiated by retrieving the whole proteome of *B. pseudomallei* under accession number (ABN48669) from the NCBI database (https://www.ncbi.nlm.nih.gov/genome/). Online server ExPasy (https://web.expasy.org/ProtParm) was used to predict the secondary structure of the protein. Protein was further screened for secondary structure prediction and disulfide bond via PSIPRED (http://bioinf.cs.ucl.ac.uk/psipred) and DIANNA (http://clavius.bc.edu/-clotelab/DiANNA).

### Predication of protein antigenicity

Antigenicity is used to predictability of proteins that bind to immune cells and provide adaptive immune responses [26]. For triggering immune cell response, antigens interact with B cells or T cells. Antigen has a biological key term as an epitope, binding to the corresponding antibodies.

Whenever T-cell receptor and MHC combine, linear amino acid sequences (epitopes) were identified. It is essential to identify the antigenic potential for each protein and potential epitopes. The protein was subjected to the online server VaxiJen for antigenicity prediction (http://www.ddg-pharmfac.net/vaxijen/VaxiJen/VaxiJen.html) [27].

### Allergenicity predication

The protein was scanned for allergic prediction by analyzing it in AllerTOP v. 2.0 (https://www.ddg.pharmfac.net/AllerTOP) online server. To properly evaluate the 3D structure trRosetta (https://yanglab.nankai.edu.cn/trRosetta), online server was used.

### Screening of T-cell epitopes and antigenicity prediction and population coverage

From the IEDB database, the common fragment was utilized for analyzing highly immunogenic T-cell epitopes via the *MHC-1* (http://tools.iedb.org/mhci/) and *MHC-2* tool (http://tools.iedb.org/mhcii/) [28]. Again, the VaxiJen v. 2.0 online server predicts MHC-1 and MHC-2 antigenicity [29]. Nonallergic epitopes were determined via AllerTOP v. 2.0 (https://www.ddg.pharmfac.net/AllerTOP). IEDB tool (http://www.iedb.org/) is used to predict population coverage for each epitope.

### B-cell epitope prediction

Predication of the most potent B-cell epitopes of proteins was confirmed via the IEDB tool. Based on allergenicity, pattern, and VaxiJen score, top epitopes were ranked.

### Epitope cluster analysis and vaccine construction

CTL, HTL, and B-cell epitopes are determined by IEDB. For vaccine construction and clusters, epitopes were utilized. Each epitope is started by B-cell and T-cell epitopes. In the host, immune interaction was induced by the interaction of Toll-like receptors and adjuvants [30]. Epitopes combine by linkers EAAAK, CPGPG, and AAY are utilized to link with all *MHC-1* and *MHC-2* epitopes, respectively. Vaccine is shown below:

EAAAKHGAEKKDEALKNDVQLGEPSWAFDEPGGGAGLNEEGRQLVRGMSRARAERDGEGCPGPGAESHVDRSIALHEHNVRLDVVFLHCPYEAHRMRYAREVVALPFLKFQYGLDTQLLLLAWRIFARLLWNPHFSVQLLAWRIFARVEVVALPFLKVLWNPHFSVAAYLPVFLHVSTDEVFQQRNPMILFRFLHVSTDIGANFVLDWLKSLQGNPKLMIANALGGYHHTYGLPVYWSALGPDAHHHHHH

### Antigenicity, allergenicity, and vaccine construct solubility predication

Via VaxiJen v. 2.0 server, antigenicity was determined. However, analysis of the allergenicity pattern of the vaccine was performed by server AllerTOP v. 2.0 (https://www.ddg.pharmfac.net/AllerTOP).

### Predication of physiochemical properties

EXPasy as an online server functionally characterizes the constructed vaccine. Isoelectric pH, hydrophobicity, instability and aliphatic index, molecular weight, in vitro and in vivo half-life, and GRAVY were predicated.

### Secondary and tertiary structure prediction

PSIPRED 3.3 (http://bioinf.cs.ucl.ac.uk/psipred/) predicts the alpha, beta-sheet, and coil structure of vaccine constructs. SCRATCH Protein Predictors determined three-dimensional (3D) structure of proteins.

### Refinement of tertiary structure

Using Galaxy Refine, the vaccine tertiary structure was refined to further modify its structure quality [30].

### Validation of structure

The constructed vaccine was validated by different online servers: PROCHECK (https://servicesn.mbi.ucla.edu/PROCHECK/) and Ramachandran plot.

### Vaccine 3D structure validation

For validation of tertiary structure, Galaxy Refine was used. If the score is more than 90, the structure is validated.

### Docking of constructed vaccine with TLR 4

Docking was performed to predict binding conformation and interactions of vaccine construct with TLR-4 by using ClusPro software. It is a widely used docking server, depending on 6 energy functions and protein types. Interacting residues were determined by PDBsum and PDBePISA.

### Codon optimization of vaccine sequence

Optimization of codons and reverse vaccination sequence are determined via JCat [31]. In this tool, 3 parameters were selected, containing restriction enzyme cleavage sites, Rho-independent transcription termination, and bacterial ribosome binding sites. JCat tool predicts GC content of vaccine sequence.

### Molecular dynamic simulation

MD simulation was performed for TLR4-vaccine complex using Amber package. Different steps of minimization, gentle heating, equilibration, and production were performed.

## Result

### Retrieval of protein sequence

The protein sequence of *Burkholderia pseudomallei* was extracted from the NCBI database in FASTA format with ID (ABN4866).

### Physiochemical characterization

The characterization of vaccine construct was performed on physical and chemical properties, and physiochemical properties of *Burkholderia pseudomallei* were demonstrated via ProtParam that recognize 251 amino acids along with 28,465.62 kDa molecular weight [32]. The pI value was 6.86. ProtParam determined value of computed instability index 23.91 which design protein is a stable one. Aliphatic index 94.06 determined that our protein is a stable one along with temperature assortment [33]. The C1305H1971N367O342S6 formula identified the number of sulfur (S), oxygen (O), nitrogen (N), carbon (C), and hydrogen (H). The value of GRAVY was −0.129.

### Analysis of secondary structure and engineering of disulfide vaccine construct

**Fig. 2:**
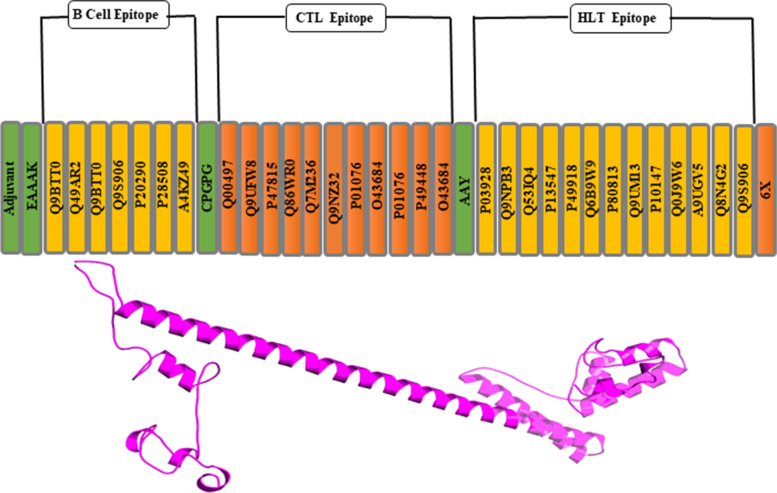
Secondary structure prediction by PSIPREDV 3.3 showing 43.5% alpha helix, 35.7% beta strand, and 20% coil

The secondary structure of the vaccine sequence was predicated on employing the online server PSIPRED [34], which exhibits 43.5% helix, 35.7% beta-sheets, and 20% loops (coil) (Fig. 2). However, disulfide bonds were demonstrated, and the score was allocated by DIANNA 1.1 tool in Table 1. By using an online server TMHMM (https://services.healthtech.dtu.dk/service.php?TMHMM-2.0), transmembrane topology was created.

**Table 1:** Predication of disulfide bond calculation by DIANA1.1

Disulfide bond scores			
Cysteine sequence position	Distance	Bond	Score
6–16	10	PKFTKCRSPER-RETFSCHWTLG	0.01456
6–42	36	PKFTKCRSPER-QEWKECPDYVS	0.01107
6–53	47	PKFTKCRSPER-AGENSCYFNSS	0.01238
6–67	61	PKFTKCRSPER-IWIPYCIKLTS	0.01052
6–81	75	PKFTKCRSPER-TVDEKCFSVDE	0.013
16–42	26	RETFSCHWTLG-QEWKECPDYVS	0.01041
16–53	37	RETFSCHWTLG-AGENSCYFNSS	0.01385
16–67	51	RETFSCHWTLG-IWIPYCIKLTS	0.01045
16–81	65	RETFSCHWTLG-TVDEKCFSVDE	0.01069
42–53	11	QEWKECPDYVS-AGENSCYFNSS	0.99779
42–67	25	QEWKECPDYVS-IWIPYCIKLTS	0.01037
42–81	39	QEWKECPDYVS-TVDEKCFSVDE	0.01063
53–67	14	AGENSCYFNSS-IWIPYCIKLTS	0.0104
53–81	28	AGENSCYFNSS-TVDEKCFSVDE	0.01213
67–81	14	IWIPYCIKLTS-TVDEKCFSVDE	0.90847

Surface-exposed residues were detected at 100–130, 151–180, and 220–250, whereas residues from 135–150, 1801–188, and 254–281 were found inside the transmembrane region. The core area of the *B. pseudomallei* was determined to have residues from 105–112, 130–135, 140–150, and 199–221.

### Analysis of antigenicity and allergenicity

Antigenicity and allergenicity were evaluated by VaxiJen 2.0 web server and AllerTOP v. 20. Value for antigenicity along with adjuvant and without adjuvant was representing that vaccine construct was antigenic. AllerTOP calculated the score, showing that the vaccine construct was nontoxic and nonallergic.

### B-cell epitopes predication

B-cell epitopes were predicted via the IEDB tool. A total of 8 numbers were selected with a 0.5 threshold value. Nontoxic, nonallergic, and antigenic epitopes were selected, and epitopes were recognized as the most effective B-cell epitopes. Ten to 50 amino acids are used to speed up the immune response; results are illustrated in Table 2. By using Kolaskar and Tongaonkar method experimentally, amino acids were predicted [35]. Analysis was performed, maximum antigenicity was 1.09, the minimum was 0.5, and the average value was 0.6. The threshold value was adjusted to 0.4, and values more than 0.4 were considered antigenic. Epitopes are used for further screening which satisfies value. The result is highlighted in Table 3.

**Table 2:** A list of Bepipred linear epitopes predicted by IEDB analysis resource

S. no	Start	End	Peptide	Length
1	7	7	H	1
2	11	19	GAEKKDEA	8
3	43	56	LKNDVQLGEPSWAF	14
4	87	88	DE	2
5	153	154	PG	2
6	305	328	GGAGLNEEGRQLVRGMSRARAERD	24
7	356	357	GE	2
8	360	360	G	1

**Table 3:** Prediction of antigenicity using the Kolaskar and Tongaonkar technique

Start	End	Peptide	Length
223	233	AESHVDRSI	10
28	36	ALHEHNVRL	9
8	16	DVVFLHCPY	9
2	10	EAHRMRYAR	9
36	44	EVVALPFLK	9
54	63	FQYGLDTQLL	10
62	70	LLAWRIFAR	9
49	57	LLWNPHFSV	9
61	70	QLLAWRIFAR	10
35	44	VEVVALPFLK	10
48	57	VLLWNPHFSV	10

Good surface acceptability is used for efficient B-cell epitopes. Emini surface accessibility was used for this analysis. A total of 0.6 was the threshold value, and based on the threshold, 12 epitopes were selected. The surface accessibility area ranged from 56 to 64 residues, although 0.57 was chosen as a minimum and 1.089 was chosen as a maximum. By using the IEDB tool, B-cell epitopes analysis was shown in Table 4. As a result, 12 epitopes were selected for vaccine construction.

**Table 4:** Prediction of Emini surface accessibility for the accessible region

Start	End	Peptide	Length
38	46	AFRFLHVST	9
26	34	AKAAFRFLH	9
1	9	EVLARGVPG	9
34	42	LIPLMIANA	9
40	48	ALGGKPLPV	9
29	37	FLHVSTDEV	9
28	36	FRFLHVSTD	9
56	64	IGANFVLDW	9
15	23	LKSLQGNPK	9
35	43	LMIANALGG	9
4	12	YHHTYGLPV	9
17	25	YWSALGPDA	9

### Predication of MHC-1 and MHC-2

 Estimation of MHC HLA alleles in humans on basis of IC50 value, via the SMM method. A lower IC50 value means a higher binding affinity of epitopes that interact with MHC-1 molecules. To maximize affinity for MH class-1 alleles, the total number of epitopes was designed to be fewer than 200. Allergic and toxic epitopes were removed having less than 0.4. For further screening, epitopes were selected.

A total number of 200 epitopes were selected, and based on antigenicity and non-allergenicity, 11 epitopes were selected. The MH class-1 epitopes were finalized, and HLA-B*40:01, HLA-B*44:02, HLA-A*02:01, HLA-A*02:06, HLA-A*02:03, and HLA-A*32:01 were dominant along with HLA-A*68:01 alleles, respectively. The epitopes LLWNPHFSV determined the highest antigenic score 2.2782.

Out of 55 epitopes for MHC-II, 12 were selected on basis of IC50 values. Epitopes were finalized on basis of toxicity, allergenicity, and antigenicity for further screening. The epitopes FLHVSTDEV and YHHTYGLPV are predicated as a higher binder that interacts with alleles (HLA-DQA1*05:01, DQB1*02:01, HLA-DRB1*04:01, HLA-DRB1*07:01, HLA-DRB1*09:01, HLA-DRB1*01:01).

### Vaccine construction

**Fig. 3:**
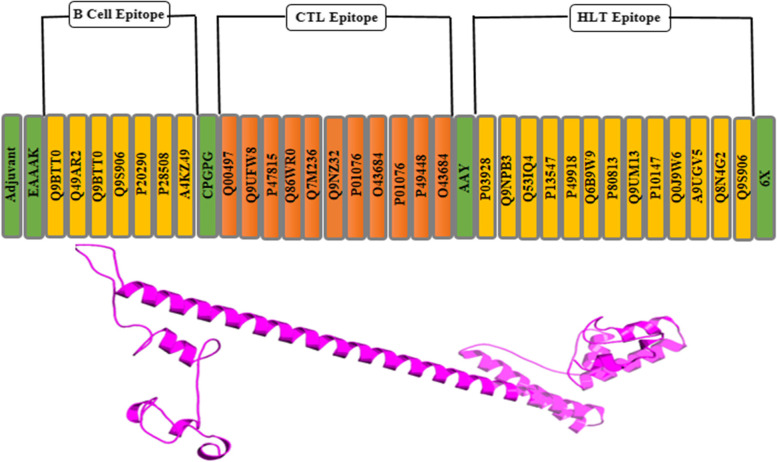
Represents vaccine construction graphical and its 3D view

The vaccine ensembles were created by combining epitopes; B-cell epitopes, MHC-1, and MHC-2 epitopes were used. 50S ribosomal protein was utilized as an adjuvant for vaccine construction. Adjuvant interacted with B-cell epitope via an EAAAK linker to create a specific immune response. CPGPG linkers were used on the linkage of MHC-1 and B cell. MH class-1 and MH class-2 epitopes were linked through AAY linkers. The B-cell epitopes, HLT, and CTL epitopes were used to merge to decrease vaccine size, and 6× His-tag was integrated at the C-terminus of vaccine sequence, for identification and purification of protein (Fig. 3). The resulting vaccine construct sequence has a molecular weight of 28,465.62 kDa and 251 aa sequence.

### Population coverage

Population coverage was performed by the IEDB tool to find out whether MHC-1 and MHC-2 alleles interacted with different epitopes. MHC-I alleles were distributed 75.5%, and MHC-II are distributed 82.82% in diverse geographic regions throughout the world. MHC-2 allele was dominant in Thailand (72%), followed by Japan, Europe, and North Africa with population coverage of 82.2, 78.5, and 83.4, respectively. The top coverage was estimated in closely related to South Asia and Norway with 79.31% and 84.79%, respectively. The lowest population coverage was estimated in Indonesia epitopes with 56.5% population coverage.

### Prediction of the tertiary structure and validation of the vaccine

**Fig. 4:**
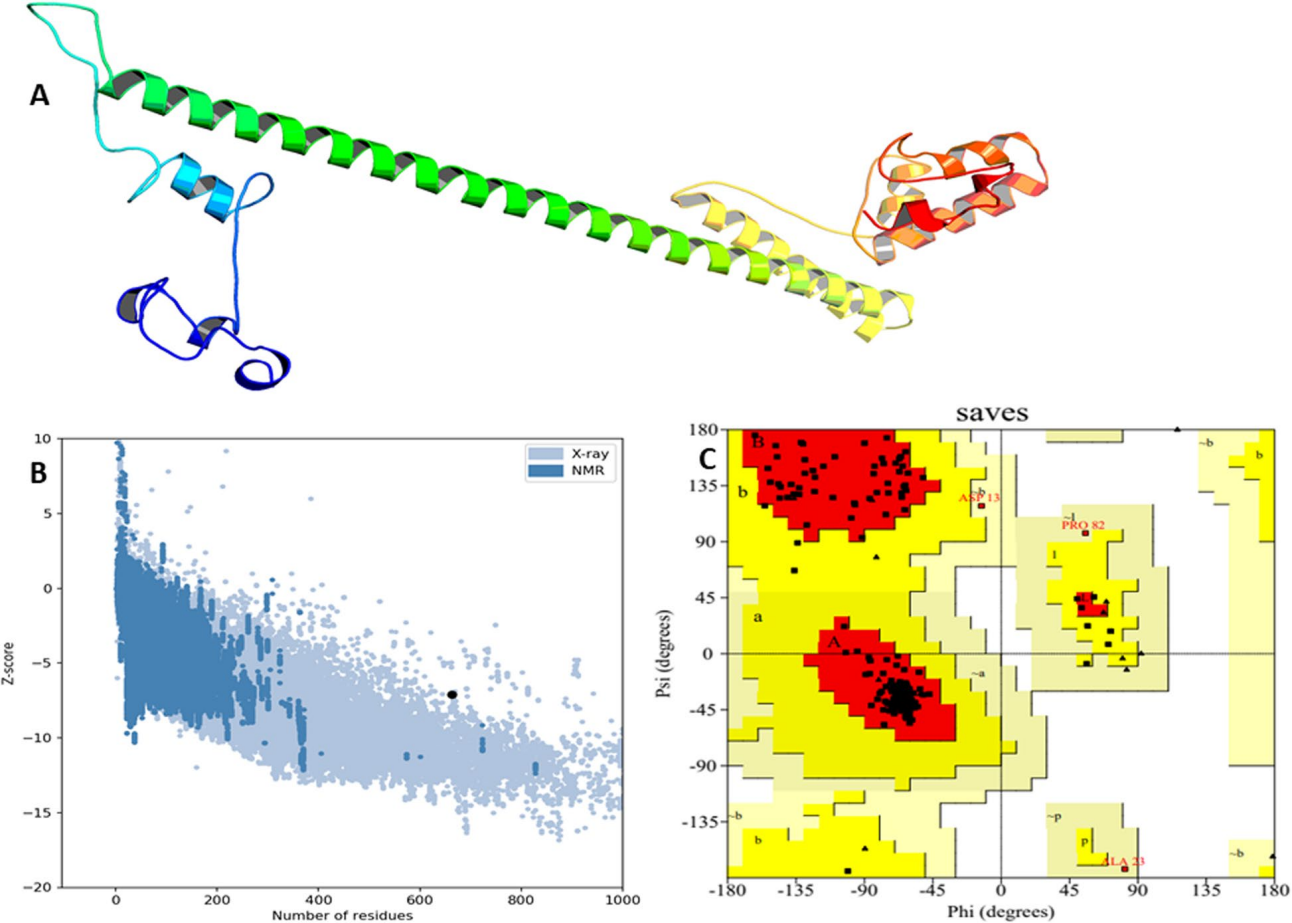
(**A**) 3D structure validation. (**B**) and (**C**) Structure validation by PROSA Web and PROCHEK

The ProSA 3D server was used to predict the 3D structure of the vaccine sequence, resulting in ten predicted structures for a given query sequence. The fifth model was taken for further investigation (Fig. 4). The ERRAT, ProSA-web, and PROCHECK services were used to validate the structure, identifying and correcting any potential mistakes in the projected tertiary structure. The ERRAT server projected the overall quality of the vaccine 3D structure, and the estimated quality score was 90.0% S. The Z-score was calculated to see if the input structure was within the range of similar-sized natural proteins. Figure 4 shows that the computed Z-score for the input structure was −8.77, indicating that it was outside the normal range for natural proteins of the same size. For Ramachandran analysis, the PROCHECK server computed 92.6% of the residues in the most favored areas, 6.0% in additional allowed regions, 1.4% in generously allowed regions, and 0.0% in residues in disallowed regions.

### Docking of vaccine construct and TLR4

**Fig. 5:**
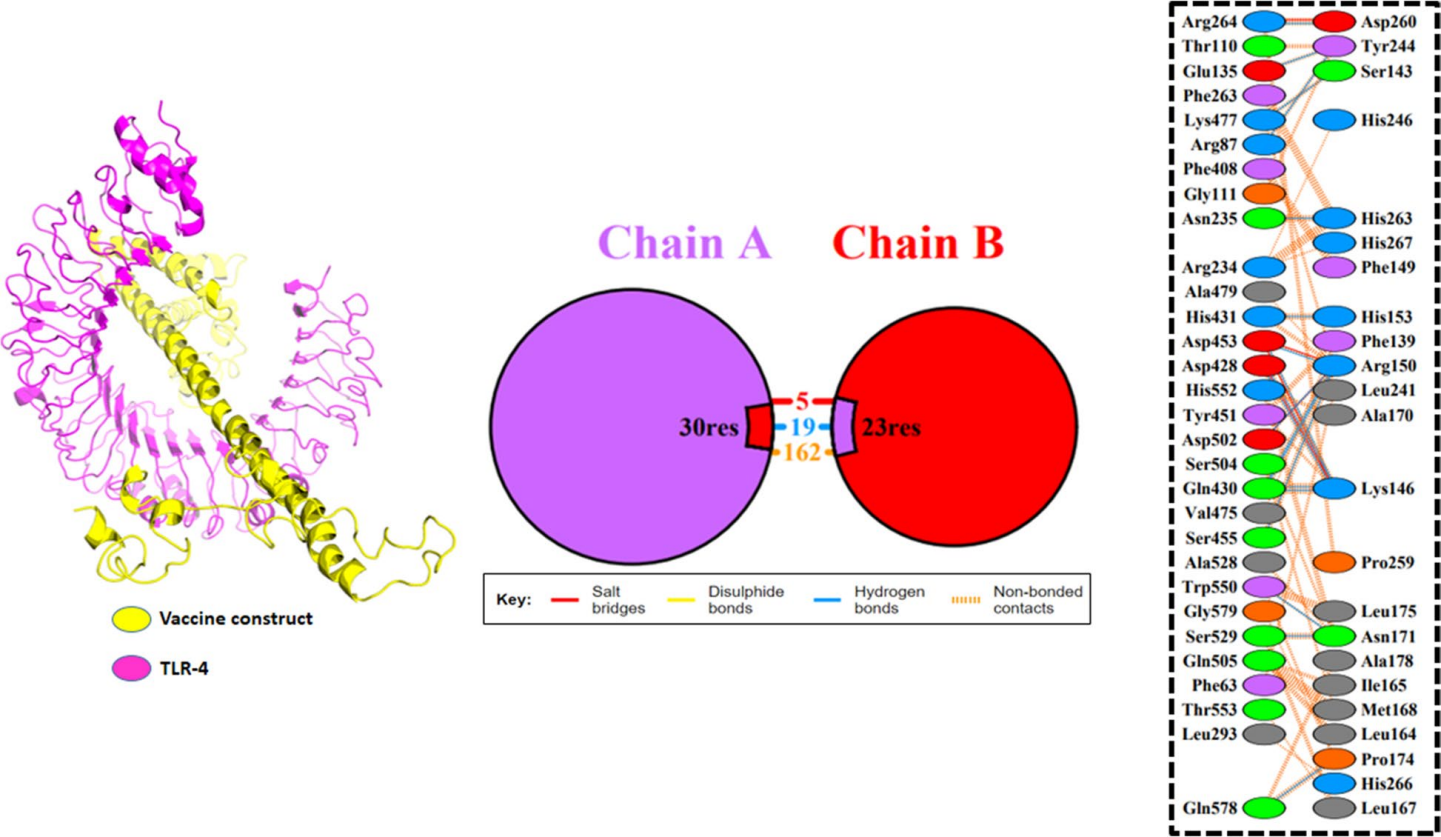
The docked complex of TLR-4 and vaccine. **A** The vaccine construct was in yellow, and TLR-4 is in purple. **B** Hydrogen bond interaction between vaccine construct and TLR-4

By using ClusPro 2.0, protein-protein docking was performed to find the interaction between vaccine construct and TLR4 [38]. A total number of 10 models were created. All complexes were analyzed through PyMOL. After analyzing different complexes, model 2 was designated for further investigation. Representation of different interactions was determined by PDBsum and PDBePISA online server. Results revealed (Fig. 5) hydrogen bonds had formed between ARG87-TYR244, GLU135-TYR244, ASN235-HIS263, ARG264-ASP260, ASP428-LYS146, ASP428-LYS146, GLN430-LYS146, GLN430-ARG150, GLN430-LYS146, HIS431-HIS153, ASP453-LYS146, ASP453-ARG150, SER455-ARG150, LYS477-SER143, ASP502-ARG150, SER529- ASN171, TRP550-ASN171, and GLN578-PRO174 with distances of 2.65, 2.64, 2.64, 2.55, 2.84, 2.54, 2.56, 2.65, 2.89, 3.07, 3.23, 3.00, 2.78, 2.70, 2.72, 2.79, 2.96, and 2.81.

### Immune simulation

**Fig. 6:**
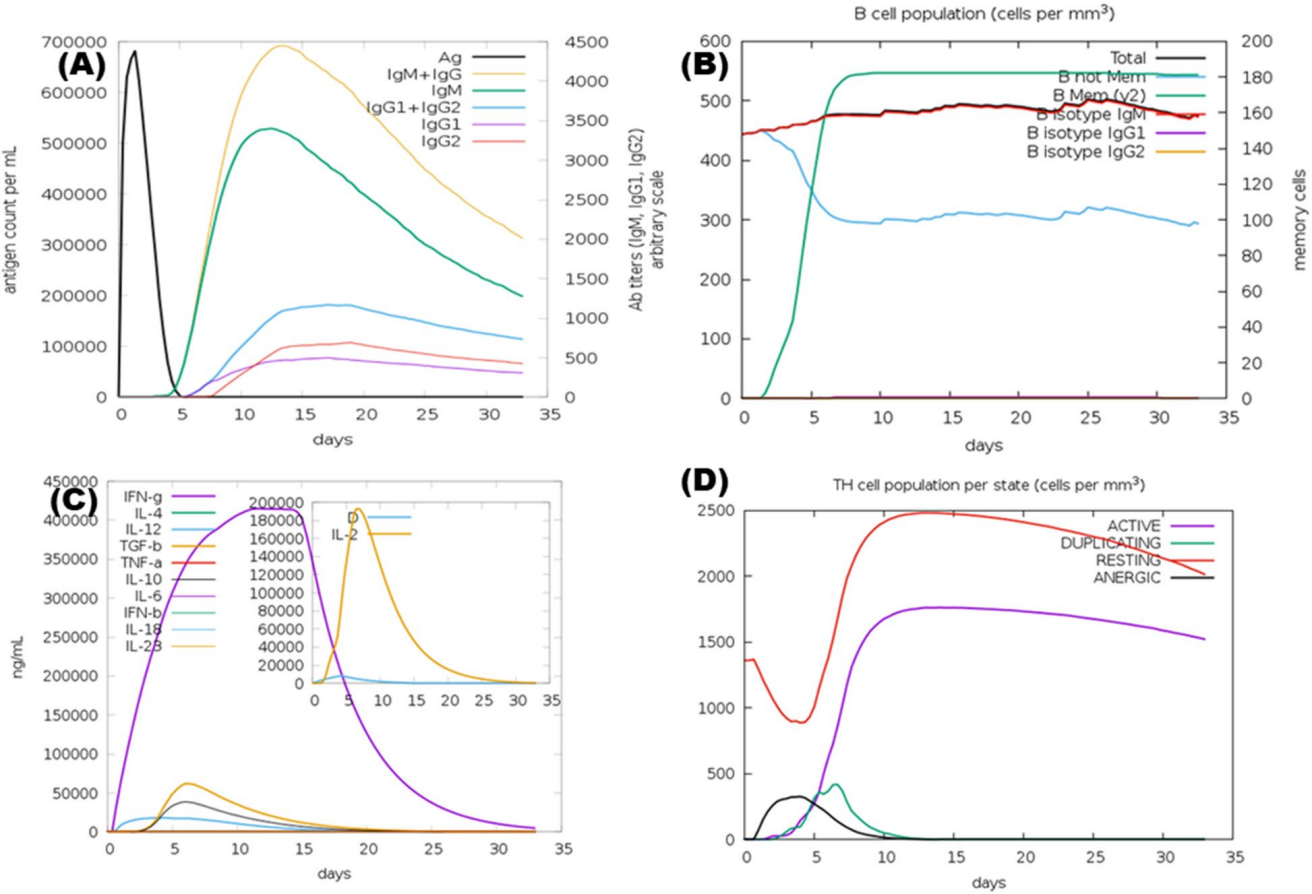
Vaccine immune simulation through C-ImmSim server. **A** Indicates the production of antibodies. **B** The population of B cell. **C** Cytokines production. **D** T-cell population

C-ImmSim server was used for immune stimulation. Figure 6 indicates that our result immune response was the same as body immune response in the human body. Figure 6A represents the production of IgG and IgM antibodies. Figure 6B shows the high level of antibody production. IFN-γ score was high as shown in Fig. 6C. TH cell population is indicated in Fig. 6D.

### Codon optimization

**Fig. 7:**
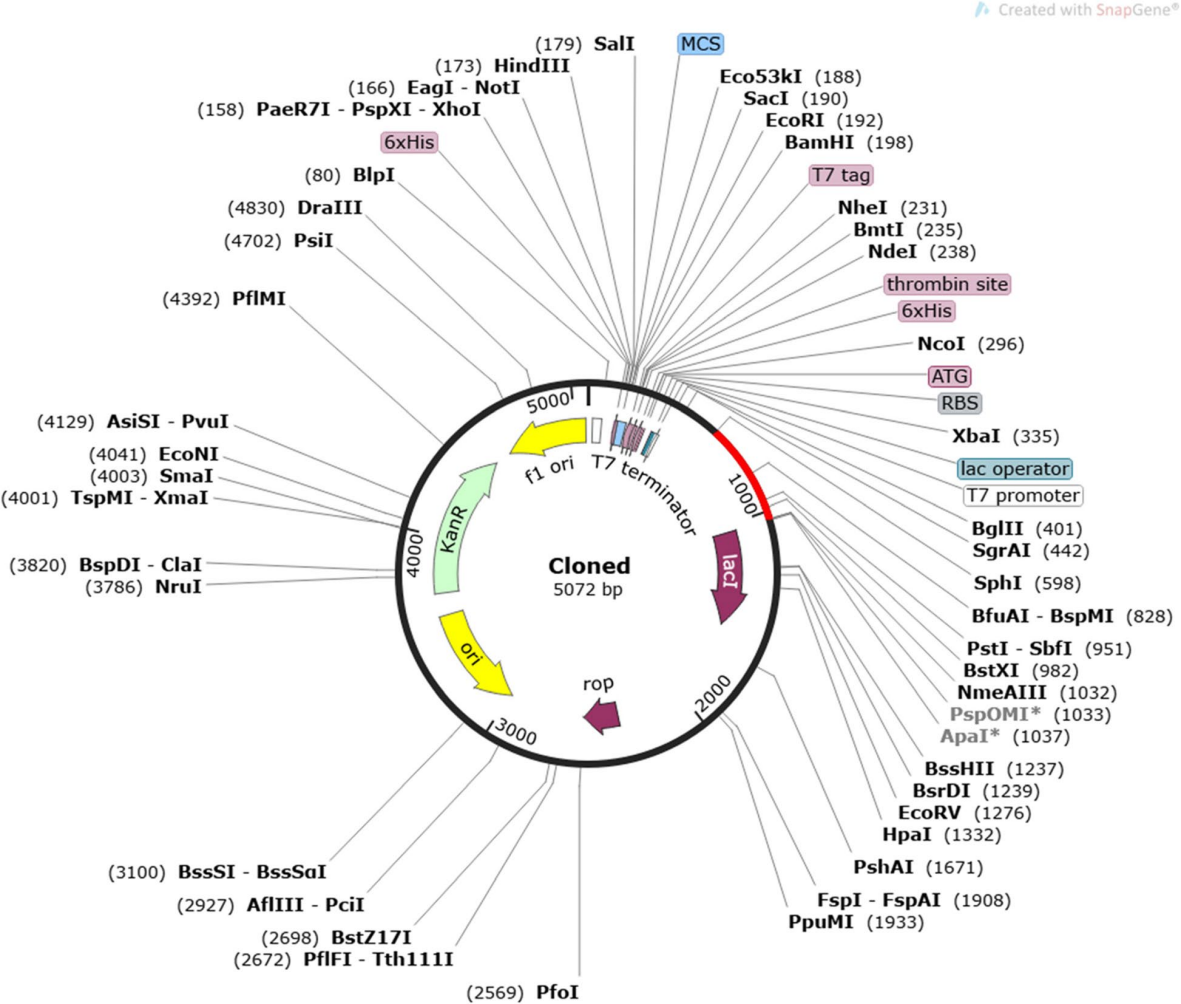
Cloning of the final vaccine where black showing vector and red showing insert

The reverse translation and codon optimization were identified in *Escherichia coli* to find expression in the vaccine through the JCat server. The vaccine sequence was comprised of nucleotides, and in sequence, CAI was 0.9541667941400593, and GC content was 70.2523240371846, which indicates expression was high. Main 2 restriction sites XhoI and NdeI were added. The restriction sites and vaccines were cloned with SnapGene software. The clone is represented in Fig. 7.

### Molecular dynamic simulation

**Fig. 8:**
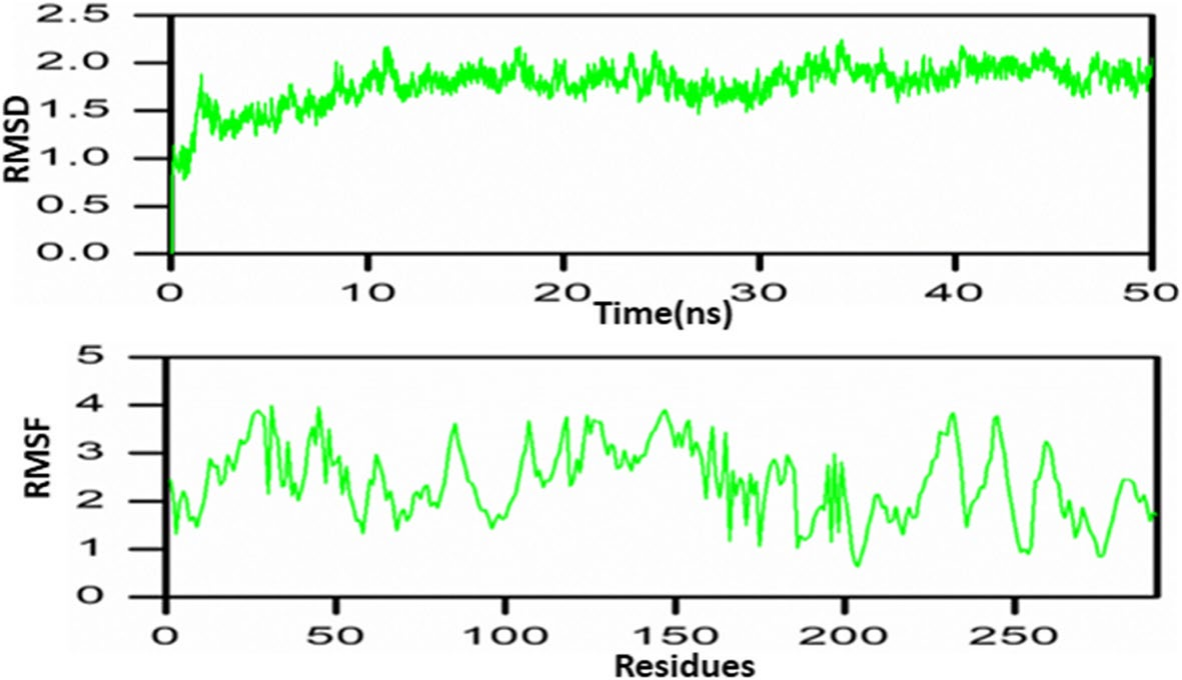
Molecular dynamic simulation showing RMSD and RMSF of the complex

TLR4 and vaccine complex stability and residual flexibility are determined by RMSD and RMSF (Fig. 8). Complex stable is still 50 ns. In the different regions, residual flexibility fluctuated.

## Discussion

Vaccination plays a vital role in immune system activation and also stops the attack of various pathogen-borne infectious disorders. The use of the surface antigenic epitopes is very crucial for designing an effective vaccine. Conventional vaccines designed for the different pathogens are used all over the world and are supposed to be the best way treatment of various disorders [36]. But these vaccines faced more problems in comparison with the in silico approach. The in silico subunit vaccines are nonhazardous, more stable, and are easily engineered compared with an old vaccine. Subunit vaccines are made up of highly immunogenic B cell and T cells which are the derivatives of the proteome of the specific pathogen [37]. For *B. pseudomallei*, up to date, no multi-epitope subunit vaccine is designed. In this study, immunoinformatics approaches were used for vaccine design against *B. pseudomallei*. First of all, proteome sequence was retrieved from NCBI.

Physiochemical properties, antigenicity, allergenicity, and secondary structures were predicated. MHC-1 and MHC-2 epitopes were also predicated.

Constructed protein antigenic score was 0.9480 at 0.5 thresholds indicating properties of antigenic vaccine and was nonallergic score at 0.5 default threshold showing nature of the nonallergic vaccine. However, the vaccine molecular weight was 28,465.62KDa. Our vaccine instability score was 23.91 which indicates the vaccine is a stable one. The theoretical pI value was 6.86, and the aliphatic index was 94.06, showing the vaccine was stable thermally. GRAVY score was −0.129, a value representing vaccine is hydrophobic. Secondary and 3D structures give information about protein function, protein-protein, protein-ligand interaction, and the dynamic of protein.

Vaccine secondary and 3D structures determined showing 43.5% alpha helix, 35.7% beta-strand, and 20.8% coil in the secondary structure were predicted. However, the 3D structure was validated via different tools such as PROCHECK, Galaxy Refine, and Ramachandran plot. All tools indicated our 3D structure vaccine is validated. ClusPro online servers were used to estimate the interaction between TLR-4 and constructed vaccines. Complex dynamics stability was confirmed by MD simulation.

## Conclusion

Present scientific research was used to design a stable and safe epitope base vaccine against *B. pseudomallei* through an immunoinformatics approach. The study starts with retrieving *B. pseudomallei* proteome from NCBI. By using immunoinformatics tools, suitable proteins were selected for effective vaccine design. Epitopes of B cell and T cell were predicated by an online server. By using linkers and adjuvant, CTL and HTL joined for vaccine construction. Physiochemical properties, allergenicity, and antigenicity were predicated and got the stable and safe vaccine. Docking was performed to find out the interaction between TLR-4 and humans. Against infection, the present scientific study was designed to make a safe, highly immunogenic, and stable vaccine by using the most reliable immunoinformatics techniques. We recommend the movement of the constructed vaccine to the biological validation phase using appropriate model organisms to validate our findings.

## Data Availability

NCBI database in FASTA format with ID (ABN4866)

## Abbreviations

MHC: Major histocompatibility complex
AMA: Apical membrane antigen
TLR: Toll-like receptor
NCBI: National Center for Biotechnology Information
IEDB: Immune Epitope Database
